# CAR T-cell therapy for systemic lupus erythematosus: current status and future perspectives

**DOI:** 10.3389/fimmu.2024.1476859

**Published:** 2024-12-19

**Authors:** Jincai Zhou, Bixia Lei, Feifei Shi, Xinran Luo, Kai Wu, Yanhong Xu, Yuting Zhang, Rongjiao Liu, Huajing Wang, Joy Zhou, Xiaowen He

**Affiliations:** Innovation & Research Department, OriCell Therapeutics Co. Ltd., Shanghai, China

**Keywords:** systemic lupus erythematosus, chimeric antigen receptor T-cell, CD19, BCMA, therapeutic strategy, clinical implications

## Abstract

Systemic lupus erythematosus (SLE) and lupus nephritis (LN) are debilitating autoimmune disorders characterized by pathological autoantibodies production and immune dysfunction, causing chronic inflammation and multi-organ damage. Despite current treatments with antimalarial drugs, glucocorticoids, immunosuppressants, and monoclonal antibodies, a definitive cure remains elusive, highlighting an urgent need for novel therapeutic strategies. Recent studies indicate that chimeric antigen receptor T-cell (CAR-T) therapy has shown promising results in treating B-cell malignancies and may offer a significant breakthrough for non-malignant conditions like SLE. In this paper, we aim to provide an in-depth analysis of the advancements in CAR-T therapy for SLE, focusing on its potential to revolutionize treatment for this complex disease. We explore the fundamental mechanisms of CAR-T cell action, the rationale for its application in SLE, and the immunological underpinnings of the disease. We also summarize clinical data on the safety and efficacy of anti-CD19 and anti-B cell maturation antigen (BCMA) CAR-T cells in targeting B-cells in SLE. We discuss the clinical implications of these findings and the potential for CAR-T therapy to improve outcomes in severe or refractory SLE cases. The integration of CAR-T therapy into the SLE treatment paradigm presents a new horizon in autoimmunity research and clinical practice. This review underscores the need for continued exploration and optimization of CAR-T strategies to address the unmet needs of SLE patients.

## 1 Introduction

Chimeric antigen receptor T (CAR-T) cell therapy is a pivotal innovation in the field of targeted immunotherapy, allowing for the direct recognition of tumor-associated antigens (TAAs) without requiring major histocompatibility complex (MHC)-mediated antigen presentation (1, 2). The CAR structure, meticulously designed to enhance the specificity and efficacy of T cell responses, consists of an extracellular antigen-recognition domain, a hinge and transmembrane region, and an intracellular signaling domain that includes costimulatory molecules (3–6). To date, numerous researchers worldwide have made significant efforts to evaluate CAR-T cells for the treatment of a broad spectrum of hematologic malignancies, including but not limited to B-cell lymphomas, T-cell lymphomas, and multiple myeloma (MM). As anticipated, the remarkable outcomes of CD19 and BCMA-directed CAR-T cell therapy in B-cell lymphoma (7, 8) and multiple myeloma (MM) (9, 10), approved by the U.S. Food and Drug Administration (FDA) and China’s National Medical Products Administration (NMPA), have substantially shifted the clinical research emphasis towards the treatment of solid tumors and non-neoplastic disorders (11).

SLE is a severe autoimmune disorder, affecting primarily women of childbearing age with a prevalence of 0.1% in the general population (12–14). The etiology of SLE is multifactorial, involving genetic susceptibility, environmental triggers, and hormonal factors; however, the precise mechanisms remain unclear (15). It is characterized by the formation of autoantibodies and immune complex deposits, leading to the destruction or dysfunction of multiple organs and affecting patients’ lifespans to varying degrees (16, 17). SLE manifests a variety of clinical symptoms, including fatigue, joint pain, skin rashes, photosensitivity, and renal inflammation (12, 18, 19). Despite advances in treatment over the past decade, which include non-steroidal anti-inflammatory drugs, glucocorticoids, antimalarial agents, and immunosuppressants, the management of SLE remains a significant challenge due to limited efficacy and adverse side effects (20–23). Given the central role of B cells in SLE pathogenesis, modulating B cell function has emerged as a key therapeutic strategy to mitigate the autoimmune response in SLE (24). Recent developments in B cell-targeting immunotherapies, such as monoclonal antibodies (mAb) against CD20 and B cell activating factor (BAFF), have shown promise in managing severe and drug-refractory SLE (25, 26). While multiple-center studies suggest the effectiveness of these therapies in treating severe and/or drug-refractory SLE, response rates among patients vary widely, and challenges such as disease progression and relapse post-treatment persist (27, 28). Hence, research increasingly indicates that achieving more effective B cell depletion and developing durable treatments for SLE patients are emerging as promising goals (24, 29).

With the advancement of innovative immunotherapies, CAR T-cell therapy holds great potential for treating patients with refractory SLE (30, 31). Numerous preclinical and clinical studies have shown the efficacy of anti-CD19 and anti-BCMA CAR-T cells in treating a range of autoimmune diseases, including SLE, by targeting CD19^+^ B cells or BCMA^+^ plasma cells or double-positive plasmablasts (32, 33). In recent years, there has been a marked increase in the registration of global clinical trials examining CAR-T therapies for SLE, with clinicaltrials.gov indicating over 30 registered trials (https://clinicaltrials.gov).

Taken together, this review aims to evaluate the potential of CAR-T cell therapy in treating SLE\-related diseases. We provide a concise summary of the main clinical advancements and potential applications of CAR-T cell therapies for SLE. Additionally, we discuss the underlying mechanisms and clinical challenges, providing valuable insights into identifying novel targets and exploring combination therapies to refine the study model and enhance clinical value.

## 2 Development of CAR-T cell therapy

To provide a foundational understanding of CAR-T cell therapy’s mechanisms and development for effective oncology application, this section offers an overview of the CAR mechanism, structural variations, and approved CAR-T products.

**Figure 1:**
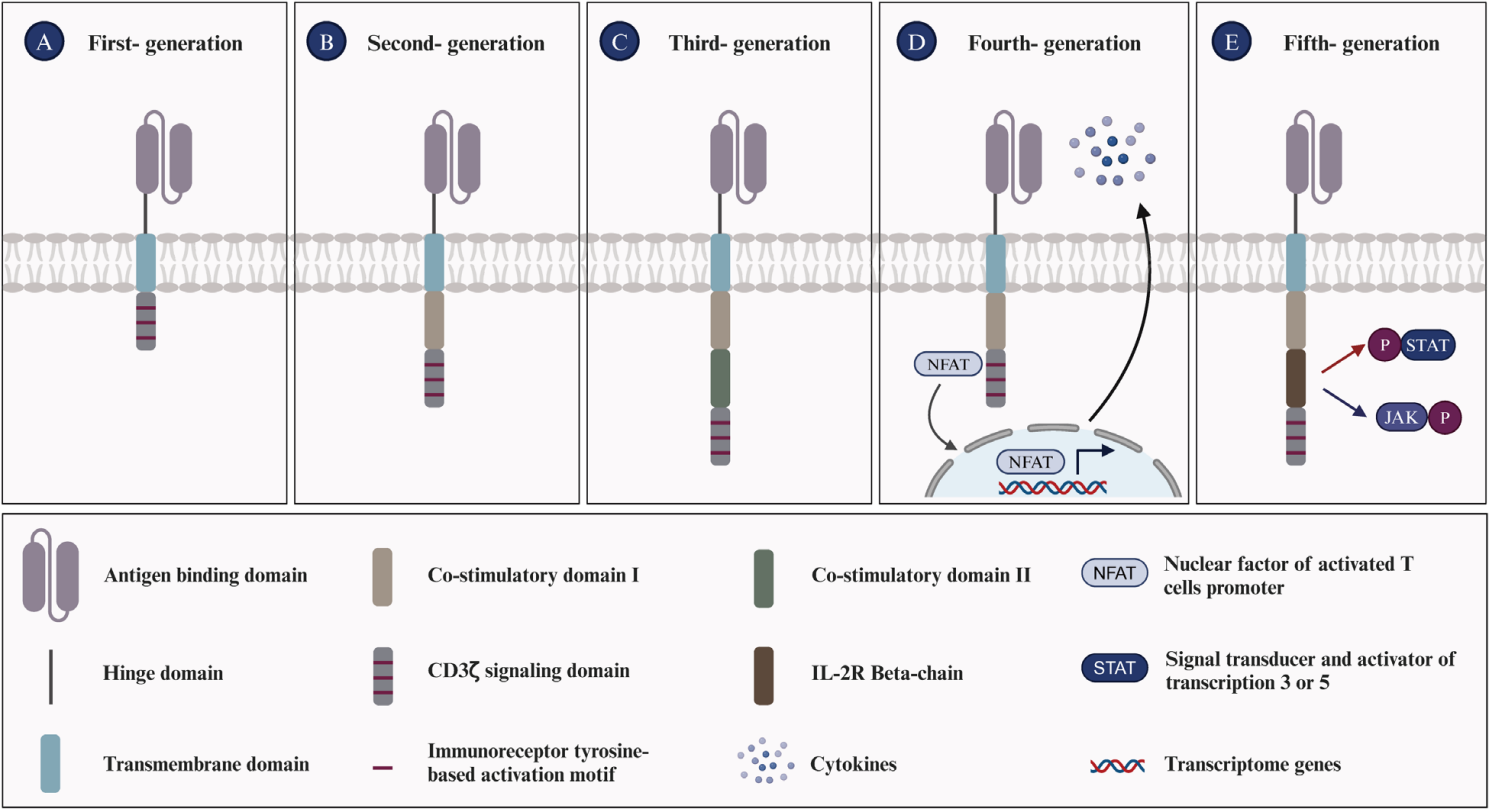
Evolution of chimeric antigen receptors T cells. **(A)** First-generation CAR-T cells are equipped with an extracellular antigen-recognizing domain and an intracellular CD3ζ domain, accounting for signal transduction. **(B)** Second-generation CAR-T cells are equipped with an extracellular antigen-recognizing domain and two intracellular domains: CD3ζ and an additional costimulatory domain (e.g., CD28, ICOS, or 4-1BB). **(C)** Third-generation CAR-T cells incorporate an extracellular antigen-recognizing domain and three intracellular domains: CD3ζ and two additional costimulatory domains. **(D)** Fourth-generation CAR-T cells, also termed ‘TRUCK’ CARs, exhibit a similar structure to second-generation CARs and feature an inducible cytokine expression profile, such as IL-12, driven by an nuclear factor of activated T-cells (NFAT)-responsive promoter. **(E)** Fifth-generation CAR-T cells integrate features from prior generations and introduce novel elements, such as the IL-2R beta-chain, which enables the activation of the JAK/STAT3/STAT5 pathway to further optimize T cell responses in an antigen-dependent manner. The evolution of CAR-T cell generations has been driven by the aim of enhancing the efficacy of cancer immunotherapy.

CAR-T cell therapy is an innovative approach to tumor immunotherapy, involving the genetic modification of T cells to express receptors that recognize tumor-specific antigens. This approach has emerged as a promising treatment strategy for a variety of malignancies. Development of CAR-T structures began in the 1990s, initially targeting B-cell lymphoma. Subsequently, the design of CAR has undergone continuous evolution and refinement (**Figure 1**) (34). First-generation CAR constructs rely on CD3ζ signaling for T-cell activation, yet lack intracellular costimulatory signals, which limits their *in vivo* expansion and persistence, impeding a durable anti-tumor response. Second- and third-generation CAR structures integrate one or more co-stimulatory molecules, such as 4-1BB (CD137) and CD28, into their intracellular domains. Others include ICOS, CD27, CD40, and OX40. These modifications are designed to enhance T-cell proliferation and survival, thereby improving clinical outcomes. Fourth-generation CAR structures, termed armored CARs, incorporate pro-inflammatory cytokines (e.g., IL-7, IL-12, IL-15, IL-18, IL-21), chemokines (e.g., CCL19, CCL21), or chemokine receptors. These modifications are designed to counteract the immunosuppressive tumor microenvironment (TME) by enhancing T-cell activity and survival, thereby directing CAR-T cells towards the tumor site. Fifth-generation CAR structures integrate a truncated cytoplasmic IL-2 receptor β-chain (IL-2Rβ) domain and a STAT3 or STAT5-binding moiety, enhancing the second-generation design. Upon activation, they can enhance the proliferation and activation of engineered T cells by promoting T cell receptor (TCR) and cytokine-driven JAK-STAT signaling (35, 36). In this therapy, T cells isolated from the patient are genetically modified to express a CAR structure, expanded *ex vivo*, and then reinfused into the patient (37). Currently, CAR-T therapy has demonstrated significant clinical efficacy, and the FDA has approved seven products for the treatment of hematological malignancies:

- Kymriah, a CD19-specific CAR-T product, was the first to be approved on August 31, 2017, for treating patients with refractory or relapsed B-cell precursor acute lymphoblastic leukemia (B-ALL) (38).
- Yescarta, a second CD19-specific CAR-T product, was approved on October 18, 2017, for adult patients with relapsed/refractory large B-cell lymphoma (R/R LBCL), including diffuse large B-cell lymphoma (DLBCL), primary mediastinal B-cell lymphoma (PMBCL), high-grade B-cell lymphoma (HGBCL), and transformed follicular lymphoma (TFL), after two or more lines of systemic therapy (39).
- Tecartus, a third anti-CD19 CAR-T product, was approved for the treatment of relapsed/refractory mantle cell lymphoma (R/R MCL) patients on July 24, 2020 (40).
- Breyanzi, another CD19-specific CAR-T product, was approved on February 5, 2021. It is indicated for the treatment of adult patients with R/R LBCL after two or more lines of systemic therapy, including DLBCL, HGBCL, and PMBCL (41).
- Abecma, the first anti-BCMA CAR-T product, was approved on March 26, 2021, and is indicated for treating adult patients with R/R MM after immunomodulatory drugs (IMiDs), proteasome inhibitors (PIs), and monoclonal antibodies targeting CD38 (42).
- Carvykti, a bi-epitope nanobody-based anti-BCMA CAR-T product, was approved on February 28, 2022, for adults with R/R MM after four or more prior lines of therapy, including IMiDs, PIs, and an anti-CD38 monoclonal antibody (10, 43). All these products are autologous CAR-T cell therapies.
- Aucatzyla, a CD19-directed CAR-T therapy, received approval on November 8, 2024, for treating adults with relapsed or refractory B-cell precursor acute lymphoblastic leukemia (BCP-ALL).

B cells and plasma cells play a crucial role in the occurrence and development of SLE. Consequently, B cell depletion offers a promising therapeutic strategy for SLE. In this context, CAR-T therapy presents an unprecedented opportunity to address the underlying immunopathology in the field of SLE by engineering T cells to specifically recognize and eliminate autoreactive B cells and plasma cells.

## 3 Immunological characteristics and pathophysiology of SLE

Understanding the characteristics and pathogenesis of SLE deepens our knowledge of this complex autoimmune disease. This section details key symptoms and crucial factors, shedding light on the potential etiological mechanisms of SLE.

**Figure 2:**
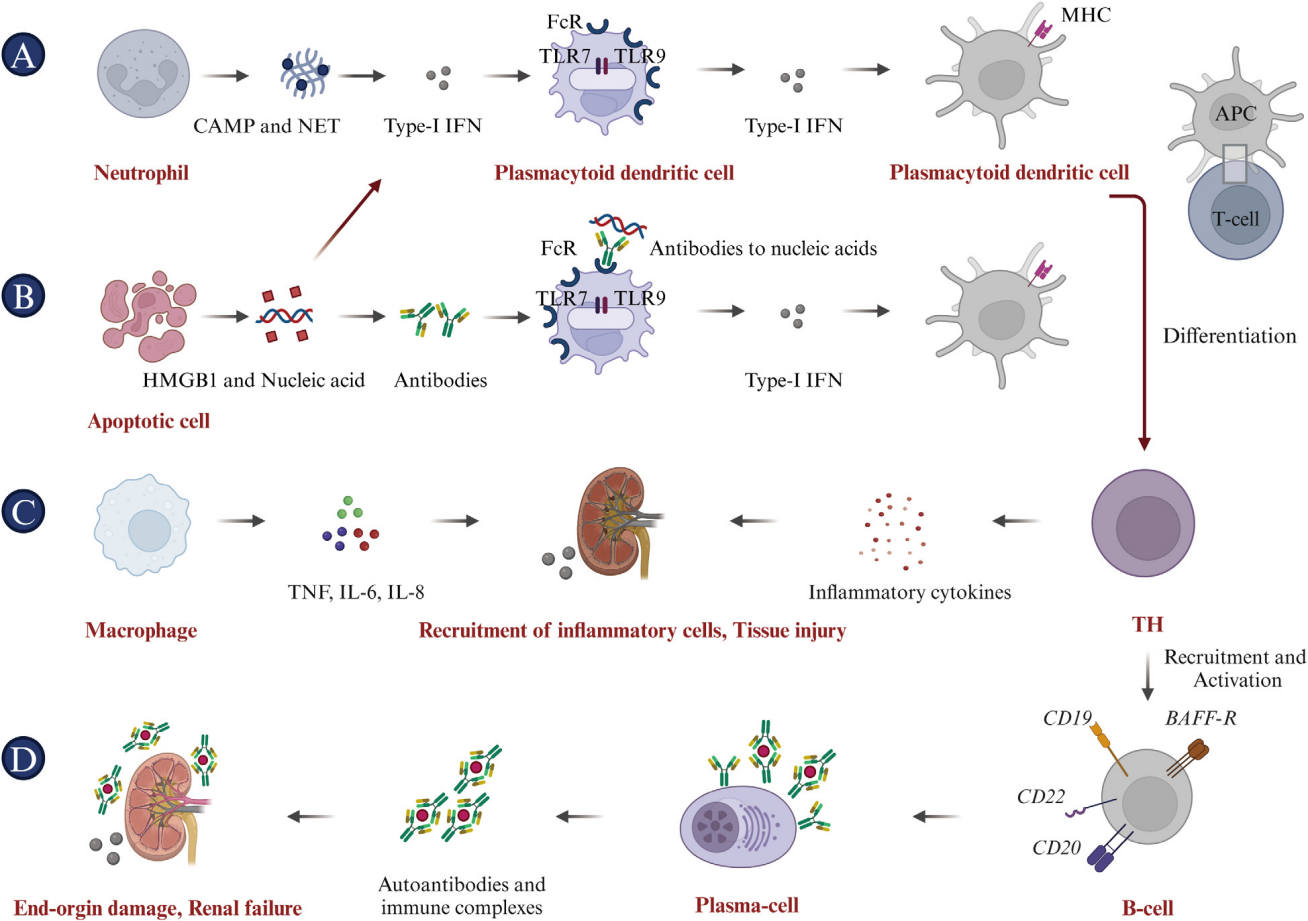
Pathophysiology of systemic lupus erythematosus (SLE). **(A)** Neutrophils initiate the pathogenic cascade in SLE by acting as key inflammatory mediators, releasing citrullinated peptides and nucleic acid antigens through NETosis, and driving Type I IFN expression. **(B)** Apoptotic cells contribute to SLE pathogenesis by providing ligands for Type I IFN expression and releasing HMGB1 and nucleic acids, promoting anti-nucleic acid antibody production and inflammatory cytokine expression. **(C)** Macrophages cause tissue damage by secreting pro-inflammatory cytokines, including TNF-α, IL-6, and IL-8. **(D)** Antigen-presenting cells (APCs), including DCs, internalize self-peptides and nuclear proteins, mature, and activate naive T cells. This leads to T-cell differentiation into Th cells and cytotoxic T lymphocytes (CTLs), activating B cells to produce autoantibodies and inducing a specific autoimmune response to nucleic acid antigens. These autoantibodies form immune complexes that cause end-organ damage, characteristic of SLE.

The immunological characteristics of SLE primarily involve dysregulated immune system activation, leading to self-tissue and organ damage. Here are some key immunological features associated with SLE: (i) Increased production of autoantibodies, including antinuclear antibodies (ANA), anti-double-stranded DNA antibodies (anti-dsDNA), anti-phospholipid antibodies, and other specified autoantibodies. These can target the body’s tissues, causing inflammation and damage (44, 45); (ii) Immune complexes form, consisting of antibodies and their antigens. These complexes can deposit in tissues, initiating an inflammatory response and resulting in damage to multiple organs (46); (iii) The increased release of inflammatory mediators, such as type-I interferons (type-I IFN), tumor necrosis factor-alpha (TNF-α) and interleukin-6 (IL-6), contributes to the inflammatory processes and tissue damage associated with SLE (47); (iv) Circulating immune complexes (ICs) in the patient’s serum trigger the complement system, leading to the depletion of significant amounts of complements C3 and C4. This consequently results in reduced levels, which are typically associated with the activity of SLE (48); (v) Impaired immune regulation, a hallmark of SLE, originates from mechanisms that fail to preserve self-tolerance. The compromised function of regulatory T cells (Tregs) and other immunoregulatory components exacerbate the autoimmune responses characteristic of SLE (49, 50). (vi) Defective efferocytosis is critical for the clearance of apoptotic particles from the body, maintaining a delicate balance among a set of “find-me”, “eat-me”, and “don’t-eat-me” signals. Notably, nucleic acids, histones, nucleosomes, and monosodium urate microcrystals serve as nuclear alarmins/”find-me” signals. Such defects can lead to a variety of diseases, including autoimmune diseases (51, 52). The pathogenesis of SLE is intricate, involving cells from both the innate and adaptive immune systems (**Figure 2**). Key immune cells, cytokines and their signaling pathways associated with SLE are as follows.

- B-cell: B lymphocytes are distinguished by the presence of the B-cell receptor (BCR) on their membrane, which is physiologically responsible for recognizing pathogens and producing specific antibodies (53). During the process of maturation, B cells may develop into autoreactive B cells, characterized by their undesired expansion and activation, which can potentially trigger SLE (54). Autoreactive B cells produce autoantibodies through underlying mechanisms, including toll-like receptors 7 & 9 (TLR7 & TLR9), which are known to effectively generate autoantibodies targeting dsDNA (55). Overexpression of TLR7 exacerbates the autoimmune response by stimulating extra-follicular B cell responses and the formation of spontaneous germinal centers (Spt-GCs) (56). Conversely, TLR9 seems to have a protective function by restricting TLR7 activity, which helps to curb these processes and underscores its regulatory role in SLE pathogenesis (57). Additionally, long-lived plasma cells and short-lived plasma blasts are also sources of autoantibody production (58). Beyond their role in generating autoantibodies that induce tissue damage, B cells also serve as antigen-presenting cells (APCs) in SLE. In this capacity, they initiate the activation of autoreactive T lymphocytes, thereby contributing to the disease’s progression (59).
- DN2 B-cell: DN2 cells, characterized by the absence of CD27, IgD, and CXCR5 expression but the presence of CD11c, are expanded in SLE patients with severe disease, including nephritis and hyperresponsiveness to TLR7 signaling, which leads to plasmablast differentiation and autoantibody production. The activation and differentiation of DN2 cells into antibody-secreting cells are driven by IL-21, produced by T follicular helper (TFH) and T peripheral helper (TPH) cells. Elevated levels of IL-21 and altered TFH/TPH activity are strongly associated with the severity of SLE (60). Moreover, BAFF signaling is critical for sustaining DN2 cells and their autoantibody production (61). Targeting IL-21, TLR7, and BAFF pathways has shown promise in modulating DN2 B-cell responses and mitigating SLE severity (62).
- T-cell: Autoreactive T cells are pivotal in the pathogenesis of SLE, as they regulate immune activation, inflammation, and target cell cytotoxicity. Upon activation, autoreactive T lymphocytes differentiate into helper T (Th) cells, which secrete a variety of pro-inflammatory cytokines and chemokines that induce immune activation and inflammation (63, 64). These cytokines and chemokines recruit and activate additional immune cells, including B lymphocytes and neutrophils, furthering the production of autoantibodies and immune complex formation (29, 65, 66). Additionally, T lymphocytes interact with dendritic cells (DC) and B lymphocytes, promoting their activation and differentiation, which culminates in the formation of germinal centers within lymph nodes—a crucial site for autoantibody production (67, 68). Moreover, T-follicular helper (Tfh) cells, found in germinal centers and extrafollicular regions, are crucial for generating autoreactive B-cell clones in SLE (69, 70). Furthermore, a higher proportion of γδ-T lymphocytes in SLE patients compared to healthy donor controls suggests their role in the autoimmune response (71, 72).
- T-reg: Regulatory T cells (Tregs) play a crucial role in cancer, autoimmune diseases, chronic inflammation, and infectious diseases by regulating self-tolerance and suppressing immune responses. Irregularities in the numbers of Tregs and/or defects in their function can lead to autoimmune diseases (73). For instance, Treg cells isolated from patients with autoimmune diseases exhibit impaired immune suppression and reduced expression of anti-inflammatory molecules like IL-10 and CTLA-4 (74). Therefore, for SLE and LN treatment, adoptive transfer of Tregs can be directly applied, either as polyclonal Tregs or as engineered variants with a receptor of high affinity for the target autoantigen, such as TCR-Tregs or CAR-Tregs, to eliminate or deactivate aberrant immune cells and reduce inflammation in AIDs (75). Doglio and colleagues isolated Tregs from the peripheral blood mononuclear cells (PBMC)s of a healthy donor, and expanded them using IL-2 and rapamycin to generate a second-generation anti-CD19 CAR. In an *in vivo* study using a humanized mouse model of SLE, it was observed that CD19 CAR-Tregs delayed the onset of B cell lymphopenia, produced immunomodulatory cytokines, showed no toxicity or reprogramming towards Th17 pro-inflammatory cells, and furthermore, in the inflamed organs, restored the normal composition of the local immune site (76).
- Neutrophils: Neutrophils are the primary cells of the innate immune system in peripheral blood circulation. Previous studies have shown that increased levels of anti-neutrophil cytoplasmic antibodies (ANCAs) in SLE patients’ serum suggest an impaired clearance mechanism for neutrophil debris, a significant source of self-antigens (77). Neutrophils can produce reactive oxygen species (ROS), leading to oxidative stress and DNA damage, thus contributing to the pathogenesis of SLE (78). On one hand, neutrophils significantly contribute to immune dysregulation and tissue damage through the formation of neutrophil extracellular traps (NETs), which are rich in decondensed nucleic acids and expel chromatin that can trigger specific autoreactive immune responses to nucleic acid antigens (79, 80). On the other hand, the enhanced formation of NETs coupled with their diminished clearance can trigger heightened inflammasome activation in macrophages, thereby intensifying the inflammatory response (81, 82). A recent study has further elucidated the correlation between neutrophil ferroptosis and SLE pathogenesis, identifying ferroptosis as the predominant mode of neutrophil death (83). The study also revealed the underlying mechanism: autoantibodies and IFN-γ in SLE serum enhance the binding of the transcription suppressor CREMα to the Gpx4 promoter, which in turn suppresses Gpx4 expression. This leads to elevated levels of intracellular lipid-reactive ROS, ultimately inducing ferroptosis in neutrophils (83).
- Apoptotic cell bodies: Lymphocytes from SLE patients frequently experience accelerated apoptosis, releasing nuclear antigens like nucleosomes into the extracellular space, which can then trigger immune responses and autoantibody formation (84). Macrophages from SLE patients often show diminished phagocytic ability, resulting in inadequate clearance of apoptotic cells (85). Furthermore, apoptotic cells in SLE often congregate in germinal centers to avoid macrophage clearance and deliver survival signals to autoreactive B cells, which may contribute to the loss of immune tolerance (86).
- Type-I IFN: Type-I IFN facilitates dendritic cell (DC) maturation, enhances autoimmune T cell survival, and activates autoantibody-producing B cells, primarily through sustained IFN-α production, autophosphorylation, and activation of the IFN-α receptor (IFNAR)-associated Janus kinases (JAKs) and signal transducers and activators of transcription (STATs) (87, 88). Numerous published studies have demonstrated a correlation between elevated Type-I IFN signatures and disease severity in both newly diagnosed and established adult and pediatric SLE patients (89–91). Type-I IFN dysregulation, observable in the majority of SLE patients, disrupts peripheral tolerance and initiates chronic activation of autoreactive lymphocytes (92). Type-I IFN suppresses the maturation of microRNA-146 via overexpression of monocyte chemotactic protein-induced protein 1 (MCPIP-1), thereby promoting unchecked inflammation and causing excessive inflammatory gene expression in SLE (93). Several clinical trials have reported promising results in SLE patients through the use of mAbs targeting IFN or IFNAR, resulting in improvements in biomarkers and clinical outcomes (94).

## 4 Current and potential treatment for SLE

**Figure 3:**
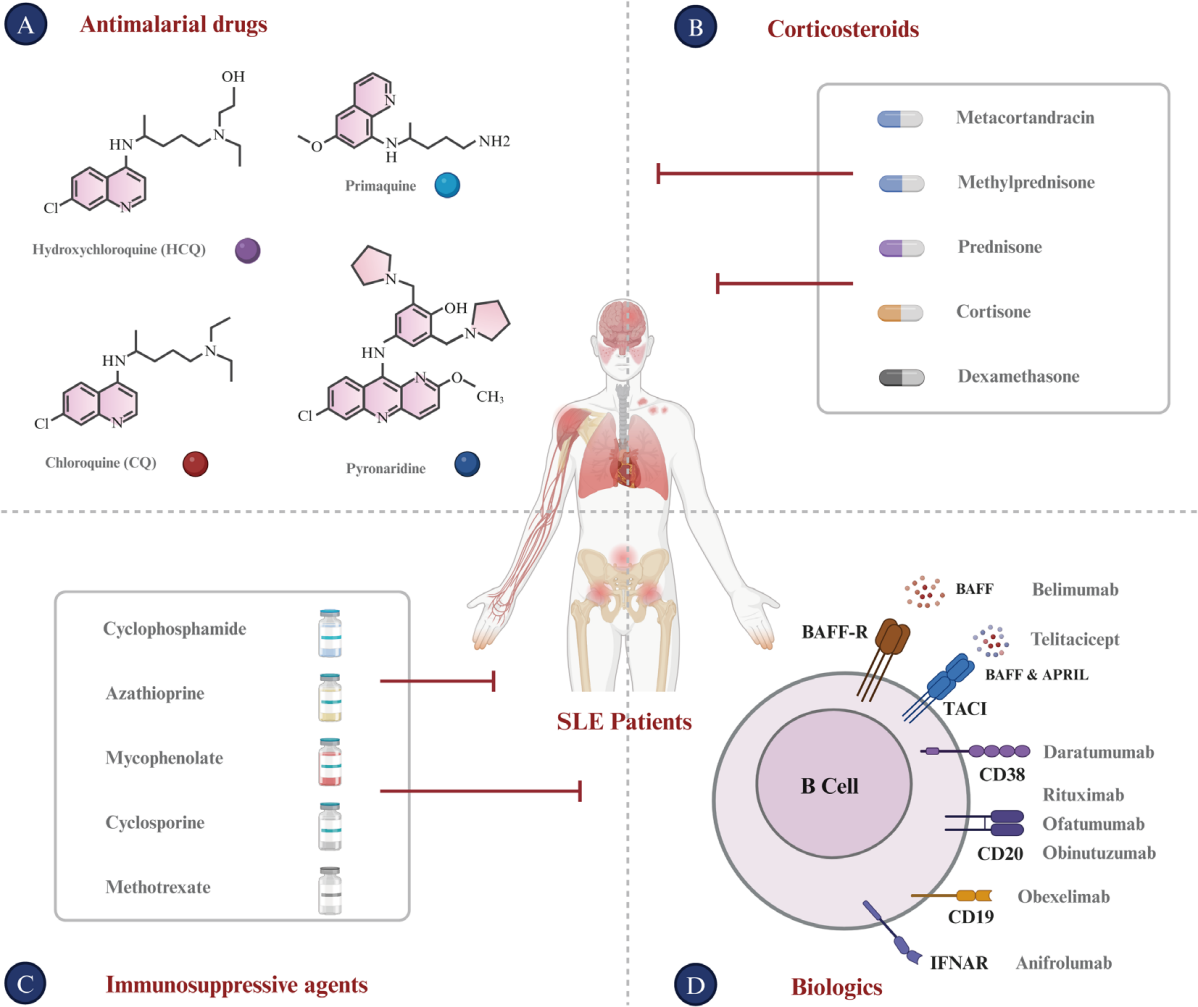
Current treatments for SLE. **(A)** Antimalarial Drugs: Chloroquine (CQ) and Hydroxychloroquine (HCQ), developed as antimalarial treatments, are now utilized for autoimmune diseases such as SLE. **(B)** Corticosteroids: Metacortandracin, Methylprednisone, Prednisone, Cortisone, and Dexamethasone are used extensively for their anti-inflammatory and immunosuppressive properties in treating SLE. **(C)** Immunosuppressive agents: Cyclophosphamide, Azathioprine, Mycophenolate, Cyclosporine, and Methotrexate are used to suppress the hyperactive immune response typical of SLE patients. **(D)** Biologics: This class encompasses monoclonal antibodies and other biologics that target specific immune system components. Monoclonal antibodies for SLE like Rituximab (anti-CD20), Ofatumumab (anti-CD20), Obinutuzumab (anti-CD20), Daratumumab (anti-CD38), and Anifrolumab (anti-IFNAR) target B-cell surface markers. Furthermore, Belimumab targets BAFF, which is expressed on B lineage cells and plays an important role in B cell proliferation and differentiation. Bi-specific antibodies for SLE, such as Obexelimab (anti-CD19xFcγRIIb) and Blinatumomab (anti-CD19xCD3), are designed to modulate B cell or plasma cell activity in SLE.

To date, there is no definitive strategy for the treatment of SLE that can replace traditional therapies (95). Current immunotherapy drugs for SLE predominantly belong to the following categories (**Figure 3**).

- Antimalarial drugs. Hydroxychloroquine (HCQ), which is known for its immunoregulatory, anti-inflammatory, antiproliferative, and anti-photoallergic properties, is commonly used to treat SLE (96, 97). The administration of HCQ may potentially reduce the risk of disease exacerbation, facilitate a reduction in corticosteroid dosage, attenuate organ damage, and counteract the thrombotic effects associated with anti-phospholipid antibodies (98). In current clinical trials, patients are recommended to use HCQ at a dosage of 5 mg/kg body weight or less in order to minimize the risk of retinal complications, even during pregnancy and breastfeeding (99). Chloroquine (CQ), known for its interference with lysosomal activity and autophagy, interacts with membrane stability and alters both signaling pathways and transcriptional activity. Accordingly, CQ’s inhibition of autophagy activity can restore the immune balance between Th17 and Treg cells in SLE, thus improving the condition (100). Additionally, HCQ and CQ can bind to nucleic acids within the endosome, preventing the interaction of endosomal TLRs with their ligands and thereby inhibiting subsequent TLR activation. HCQ and CQ exert their immunomodulatory effects by inhibiting TLR7 and TLR9 signaling, which plays a crucial role in the pathogenesis of autoimmune diseases such as SLE (101–103). However, it is important to note that while the effects of HCQ and CQ on TLR signaling are well-documented, they are not believed to be the sole mechanisms of these drugs. Multiple studies have demonstrated that consistent administration of HCQ and CQ during the first five years of the disease improves treatment outcomes, whether used as monotherapy or in combination with other therapies (104). Furthermore, long-term follow-up studies have demonstrated their safety, efficacy, and diverse therapeutic benefits.
- Corticosteroids. In addition to antimalarials, corticosteroids such as prednisone and dexamethasone are frequently used in the management of SLE. They possess potent anti-inflammatory and immunosuppressive properties, which can effectively regulate the activity of SLE and relieve associated symptoms (105, 106). Additionally, corticosteroids can also interfere with the antigen-presenting process and down-regulate the expression of MHC molecules (107). Long-term corticosteroid use in SLE is significantly associated with permanent organ damage, including osteoporotic fractures, diabetes, cataracts, avascular necrosis, and symptomatic coronary artery disease (108). Higher cumulative doses and high-dose regimens are associated with increased disease duration and risk of damage. Therefore, corticosteroid therapy for SLE requires tailored strategies and close monitoring to balance benefits and manage potential adverse effects. Steroid-sparing treatments, including immunosuppressive agents and biologics such as hydroxychloroquine, can reduce dependence on corticosteroids and limit their associated damage (109).
- Immunosuppressive agents. Cyclophosphamide, azathioprine, methotrexate, and mycophenolate inhibit lymphocyte proliferation and activation, thereby curtailing the immune response (110, 111). The administration of these agents significantly reduces the B cell population and suppresses autoantibody production (110). These agents selectively modulate antibody production, reducing IgG and IgM levels while enhancing IgE production (112, 113).
- Biologics. Rituximab, a monoclonal antibody targeting the CD20 antigen, depletes CD20-positive B lymphocytes through antibody-dependent cell-mediated cytotoxicity (ADCC), complement-mediated cytotoxicity (CDC), antibody-dependent cellular phagocytosis (ADCP), and programmed cell death (PD), providing a robust rationale for its use in treating SLE (114, 115). In addition, rituximab may also influence the function and survival of other immune cells, cytokine secretion, and immune cell movement. These effects may contribute to its therapeutic effect in SLE (116). A recent study involved 40 Rituximab-treated patients with a median age of 14.3 years at diagnosis. Post-treatment, the Systemic Lupus Erythematosus Disease Activity Index (SLEDAI) 2000 score declined from 8 to 4 over a two-year period, and levels of complement components C3 and C4 returned to normal. Moreover, anti-double-stranded DNA (anti-dsDNA) levels normalized within six months. Ultimately, 8 patients (20%) achieved disease control, and 35 (87.5%) experienced no flare-ups over a median 2-year follow-up, suggesting improved disease management (117). However, in several Phase III trials, Rituximab failed to meet its primary efficacy endpoints. The EXPLORER study, which focused on moderately-to-severely active extrarenal SLE, found no significant difference between Rituximab and placebo across primary and secondary endpoints (25). Additionally, the LUNAR trial, targeting LN, also failed to show meaningful efficacy differences (118). The lack of success at Phase III was attributed to factors such as high baseline immunosuppressive therapy in patients and complex disease mechanisms in SLE, potentially limiting the application of Rituximab as a monotherapy in SLE (119). These failed trials highlight the need for tailored trial designs and novel therapeutic approaches in future SLE research. Ofatumumab, a fully human monoclonal antibody that targets CD20^+^ B cells, has demonstrated inhibitory effects on B lymphocyte activation (120). A single-center, retrospective case series assessed the impact of ofatumumab on B cell depletion in 16 SLE patients who had rituximab allergies. B cell depletion was achieved in 12 patients, and there were improvements in complement and autoantibody levels. Additionally, ofatumumab elicited complete or partial responses in 50% of patients, indicating its potential as an alternative for those with rituximab allergies (121). Obinutuzumab, a fully humanized type II anti-CD20 monoclonal antibody that can induce extensive B-lymphocyte depletion, has been evaluated in clinical trials for SLE, particularly focusing on LN (122). The NOBILITY trial, a Phase II study, demonstrated that 35% of patients in the obinutuzumab group achieved Complete Renal Response (CRR) at week 52, compared to 23% in the placebo group, although this difference was not statistically significant (P = 0.115). By week 76, the CRR rates were 40% for the obinutuzumab group versus 18% for the placebo group, representing a statistically significant difference (P = 0.007) (123). Building on the Phase 2 results, the REGENCY trial, a Phase III study, announced in September 2024 that it met its primary endpoint. A higher percentage of patients treated with obinutuzumab plus standard therapy achieved CRR at 76 weeks compared to those receiving standard therapy alone. Furthermore, key secondary endpoints, including the percentage of patients achieving CRR with successful reduction of corticosteroid use and improvement in proteinuric response, were also met. The safety profile of obinutuzumab remained consistent with previous findings, and no new safety signals were identified. Obexelimab, a humanized high-affinity monoclonal antibody capable of binding simultaneously to CD19 and FcγRIIb, effectively inhibits the activation and proliferation of B cells, plasmablasts, and plasma cells in SLE patients (124). A recent study enrolled 104 patients, randomly assigned to receive either obexelimab or placebo. Despite lack of statistically significant outcome, 42.0% of patients treated with obexelimab (21/50) showed improvement compared to 28.6% on placebo (12/42) (P = 0.183). Overall, treatment with obexelimab led to an approximate 50% reduction in B cells (125). In addition to CD19 and CD20, B-cell activating factor (BAFF) is essential for B-cell survival and maturation. By inhibiting BAFF, Belimumab reduces abnormal B-cell activity, a central feature of SLE. Clinical trials, such as BLISS-52 and BLISS-76, demonstrated that Belimumab decreases disease activity and flare rates, improving clinical outcomes in patients with SLE (126). Belimumab has demonstrated a favorable safety profile with minimal risk of adverse effects, even with prolonged use up to 13 years, making it a suitable alternative to traditional immunosuppressive therapies (127). Belimumab received FDA approval in 2011 for treating moderate to severe SLE in individuals aged 18 and older (128). Telitacicept, a transmembrane activator and cyclophilin ligand interactor (TACI)-Fc fusion protein, has shown significant clinical effects in the treatment of immunoglobulin A nephropathy (IgAN) as demonstrated in a phase II randomized placebo-controlled trial. Patients receiving telitacicept, particularly at a dosage of 240 mg weekly, experienced a substantial reduction in proteinuria, with a mean decrease of 49% from baseline, which was statistically significant compared to the placebo group (P = 0.013). Importantly, telitacicept treatment resulted in continuous reductions in serum IgA, IgG, and IgM levels, indicating its immunomodulatory activity (129). Furthermore, adverse events were similar across all groups, with treatment-emergent adverse events being mild or moderate, and no severe adverse events were reported. On March 12, 2021, Telitacicept received its first approval from the NMPA in China for the treatment of patients with SLE (130). Anifrolumab, a mAb that binds to IFNAR and inhibits the activity of all type-I IFNs by blocking their binding, is the sole anti-IFN therapy that have undergone Phase III randomized controlled trials (RCTs) (94, 131). In July 2021, Anifrolumab received FDA approval in the USA for treating adult patients with moderate to severe SLE who are on standard therapy. Clinical studies are ongoing globally, and Anifrolumab is under regulatory review in the EU and Japan (132). The emergence of Anifrolumab underscores the potential for targeting type-I IFN in the treatment of SLE.

Additionally, Yiting Chen and colleagues have reported that the combination therapy of rituximab and belimumab demonstrates enhanced efficacy in treating SLE (133). The study’s findings are evidenced by a complete renal response (CRR) in 12 patients (66.7%) and an overall response (OR) in 13 patients (72.2%) within the combined treatment group.

New biologics and targeted therapies in SLE target a broad spectrum of key immune players, including B/T lymphocytes, plasmacytoid dendritic cells (pDC), plasma cells, T/B cell co-stimulation molecules, cytokines or their receptors, and various intracellular signaling pathways. Here, we summarize the promising therapeutic targets for SLE therapy, which align with recent developments in understanding the disease’s pathogenesis and the diversification of the SLE drug pipeline.

- BAFF-R: B-cell activating factor receptor (BAFF-R, also known as BR3) is a type III transmembrane protein encoded by the TNFRSF13C gene. It specifically binds to BAFF (TNFSF13B), promoting the survival and activation of mature B-cells (134). BAFF and BAFF-R are essential for regulating the proliferation and survival of B cells, including autoreactive ones, during their development (135). Additionally, BAFF-R enhances CD19 expression in B cells via the NF-κB pathway, amplifying BCR signaling and p100 production (136). Ianalumab, a fully human monoclonal antibody targeting BAFF-R, is under investigation in a randomized, double-blind phase III trial to assess its efficacy and safety in SLE patients (ClinicalTrials.gov Identifier: NCT05624749) (137). This highlights the promising potential of anti-BAFF-R CAR-T therapy as an appealing therapeutic approach for SLE.
- TACI: Transmembrane activator and cyclophilin ligand interactor (TACI), a lymphocyte-specific member of the TNF receptor superfamily (TNFRSF13B), is predominantly expressed in mature B cells (138). By engaging with BAFF and APRIL, TACI activates signaling pathways essential for plasma cell differentiation and survival, contributing to the pathogenesis of autoimmune diseases like SLE (139). Moreover, an enhanced TACI signal may drive the conversion of autoreactive IgM antibodies to IgG, potentially aggravating SLE (139). Consequently, TACI presents a promising therapeutic target for SLE, offering the possibility to mitigate symptoms through inhibition (140).
- CD22: CD22 (also known as Siglec-2) is a receptor belonging to the immunoglobulin superfamily, predominantly expressed on immature and mature B cells, but not on plasma cells (141). CD22 plays a crucial role in regulating B cell activation, proliferation, and differentiation. Since 2015, the humanized anti-CD22 monoclonal antibody epratuzumab has been investigated for the treatment of SLE, holding promise for patients with moderate to severe active disease (142–144). Thus, modulating CD22 activity is proposed as a therapeutic strategy to alleviate SLE-associated symptoms.
- CD40: CD40, a member of the tumor necrosis factor receptor superfamily, acts as a receptor on B cells to mediate interactions between B and T cells (145). It can activate B cells and trigger multiple pathways that enhance their survival, proliferation, and development (146, 147). Research indicates that CD40 likely plays a critical role in inducing the production of autoantibodies and immunoglobulins by autoreactive B cells, which in turn contributes to the pathogenesis of SLE (148). A case-control study found that SLE cases exhibited a statistically significant higher expression of CD40 than controls (p < 0.001), with the number of CD40-positive B cells declining significantly after remission (149). Considering the upregulated CD40 in SLE patients, targeting this receptor is a promising therapeutic strategy for modulating immune responses in SLE.
- CD70: CD70, a type II transmembrane glycoprotein and member of the TNF ligand family, is expressed on activated T and B lymphocytes, but not on resting cells (150). It interacts with CD27 to mediate immune cell activation and survival, implicating it in SLE pathogenesis (151, 152). Theoretically, autoreactive T cells are expected to stimulate B cell proliferation, differentiation, and survival, exacerbating SLE symptoms caused by autoreactive B cells. Repression of the CD70 molecule and its signaling pathways can effectively prevent T and B cell interaction, thereby ameliorating SLE (153). Consequently, CD70 presents itself as a promising therapeutic target for SLE.
- CD79b: BCR signaling is crucial across various stages of the B cell life cycle (154). Structurally, the BCR comprises a membrane-bound immunoglobulin (Ig) molecule noncovalently linked to CD79a (Igα) and CD79b (Igβ), which are transmembrane signaling subunits (155). CD79b is predominantly expressed on the cell surface of pre- and mature B cells in the bone marrow, facilitating B cell progression and maturation. It is essential for the development and maintenance of mature B cells (156). Additionally, CD79b contains ITAMs in its cytoplasmic domain, such as Y195 and Y206, crucial for transmitting BCR signals that are vital for B cell development and activation in response to antigens (154, 157). Previous studies have shown that targeting CD79b can inhibit B cell activation and reduce autoantibody levels in animal models (158). Consequently, CD79b presents itself as a compelling target for antibody-based therapies and CAR T-cell strategies within the realm of autoimmune disorders. These insights could inform the development of therapeutic strategies targeting CD79b for SLE.
- CD38: CD38 is a type II transmembrane glycoprotein belonging to the NAD+ hydrolase enzyme family (159). This receptor is widely expressed in diverse immune cells, including T and B lymphocytes, natural killer cells (NK), macrophages, and dendritic cells (DC) (160). CD38 plays a pivotal role in the activation, proliferation, differentiation, and apoptosis of immune cells, and its expression levels undergo alterations in various pathological conditions such as autoimmune diseases and cancer (160, 161). A clinical study titled “Targeting CD38 with Daratumumab in Refractory Systemic Lupus Erythematosus” found that Daratumumab treatment elicited clinical and serological responses in two patients with treatment-resistant SLE. This treatment promotes the depletion of autoreactive long-lived plasma cells and correlates with decreased interferon type I activity and modulated effector T-cell response (162). Based on these findings, CD38 has become an attractive therapeutic target for SLE.
- CD138: CD138, also known as syndecan-1, is a type I transmembrane heparan sulfate proteoglycan and a member of the syndecan family. It regulates various biological responses by interacting with chemokines, cytokines, growth factors, and adhesion molecules (163). CD138 is primarily expressed in plasma cells derived from B cells and is implicated in the pathogenesis and progression of SLE. Animal studies suggest that CD138 expression on T cells contributes to lupus progression in MRL/lpr mice (164). In individuals with SLE, CD138-positive T cells with a TCM phenotype rapidly activate autoreactive B cells, enhancing disease progression and autoantibody production in murine SLE models (165). A clinical study also revealed a higher number of CD138^+^ plasma cells in the kidneys of lupus nephritis patients, indicating their potential role in renal damage in SLE (166). These findings suggest that targeting CD138 could be a promising therapeutic strategy for active SLE patients.

## 5 CAR-T cell therapy for SLE and target selection

Conventional drugs discussed earlier have significantly improved the survival rate for individuals with SLE. Nevertheless, these treatments can often result in severe side effects and disease relapse following discontinuation (20, 167–169). Developing new treatments with comparable or higher efficacy, lower toxicity, and fewer complications is essential. Over the last decade, T cell-based therapies have gained attention for their potential in long-term SLE management and possibly a cure (170, 171). Pre-clinical studies of CAR-T cells in mice have demonstrated promising outcomes against SLE (172, 173). Monoclonal antibodies targeting B cell antigens, such as type-I and type-II anti-CD20 antibodies, have demonstrated varying degrees of efficacy in treating B cell-mediated diseases. Type-I anti-CD20 antibodies, such as rituximab, often rely on ADCC and CDC for B cell depletion, but this approach may not be as effective as anticipated due to the heterogeneity of B cell populations and the ability of some B cells to evade these mechanisms. Type-II anti-CD20 agents, such as obinutuzumab, have been associated with higher response rates due to their enhanced ability to induce direct B cell apoptosis and their prolonged serum half-life, potentially leading to a more sustained therapeutic effect. Compared to monoclonal antibodies, CAR-T cells demonstrate an enhanced capability to migrate to tumor sites and reside within the tumor microenvironment, potentially enhancing their therapeutic potential.

**Figure 4:**
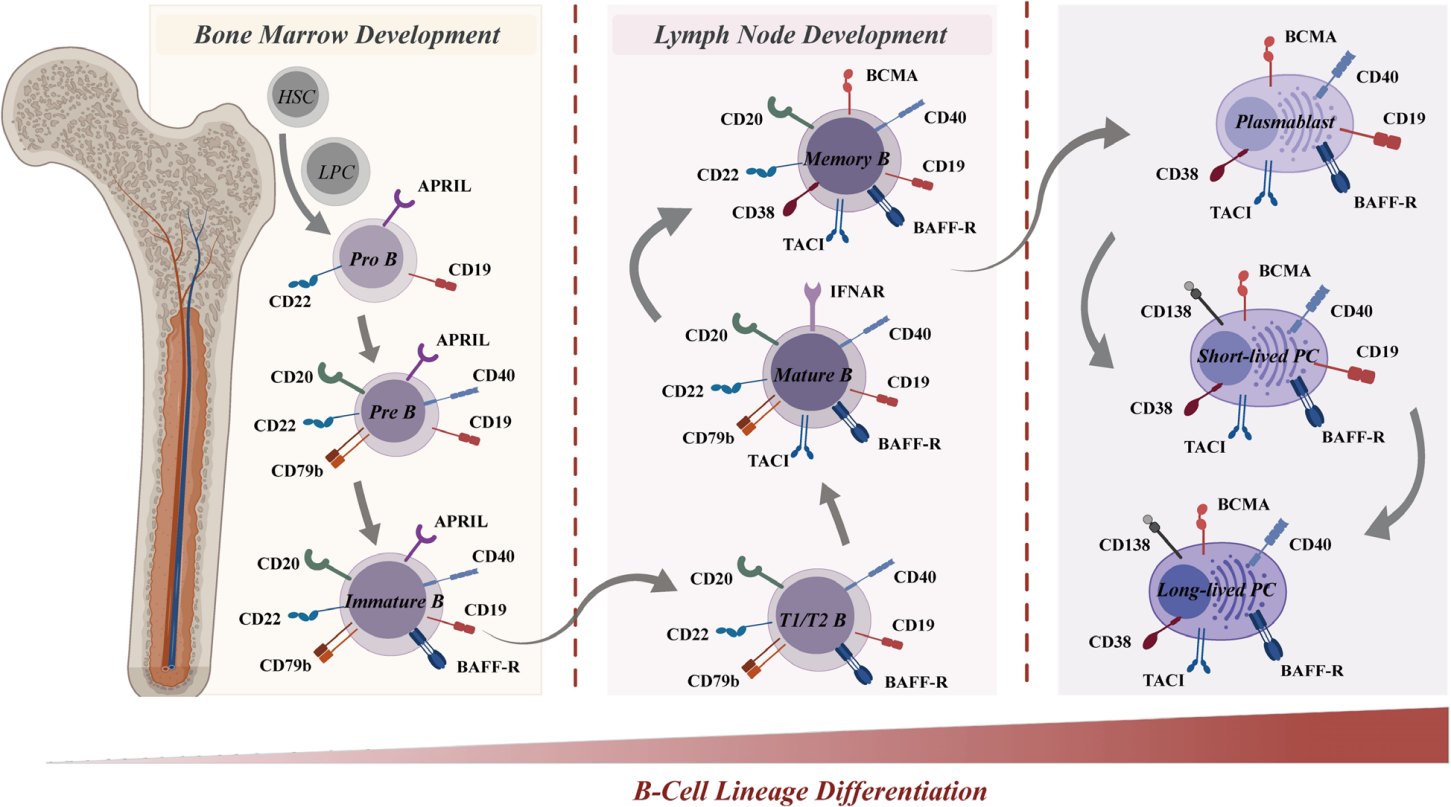
B-cell lineage differentiation and surface antigen expression in the immune system. B-cell lineage differentiation, from the bone marrow (left) to the lymph node (right), is crucial for the immune system and involves the sequential development of pro-B cells, memory B cells, and plasma cells. The differential expression of surface markers allows for the identification of various B-cell stages, from pro-B cells to plasma cells. Surface antigens, such as CD19, CD20, CD22, BAFF-R, and CD40, are represented at various stages, highlighting their roles in B-cell development and function. Other molecules, including APRIL, CD79b, TACI, BCMA, CD38, and CD138, are also depicted, underscoring their importance in B-cell and plasma cell activation and survival. The distinct expression profiles of these antigens serve as a guide for selective targeting in CAR-T cell therapies. BAFF-R, B-cell activating factor receptor; APRIL, A proliferation-inducing ligand; TACI, T-cell-activating and cytokine receptor; BCMA, B-cell maturation antigen.

Given that CAR-T cells predominantly target the B-cell lineage in SLE treatments, this section presents a systematic investigation into B-cell differentiation and surface antigen expression, aiming to enhance our understanding of unique role of B cells in SLE pathogenesis and antigen selection for CAR-T cell therapy for SLE (**Figure 4**).

The development of B cells starts with progenitor B cells in the bone marrow, which then undergo a series of proliferation and differentiation steps, transforming the early pro-B cells (CD19^+^/CD22^+^) into pre-B cells (CD19^+^/CD20^+^/CD22^+^/CD40^+^/CD79b^+^) (174). Upon further differentiation into immature B cells (CD19^+^/CD20^+^/CD22^+^/CD40^+^/CD79b^+^/BAFFR^+^) in the bone marrow, these cells undergo antigen receptor gene rearrangement to generate a unique B cell receptor (BCR) on their surface during the subsequent stage of differentiation. The BCR facilitates the specific binding of immature B cells to antigens, triggering their activation and subsequent proliferation and differentiation. Subsequently, activated B cells migrate to lymph nodes or other secondary lymphoid tissues for affinity maturation (175). In this process, B cells with high-affinity binding to antigens are favored for survival and differentiation into mature B cells (CD19^+^/CD20^+^/CD22^+^/CD40^+^/CD79b^+^/BAFFR^+^/TACI^+^). Eventually, mature B cells emerge that express surface immunoglobulin (Ig) and possess the capacity to generate antibodies. Depending on the nature of the immune response, these mature B cells can differentiate into various cell types, including memory B cells (CD19^+^/CD20^+^/CD22^+^/BCMA^+^/CD38^+^/BAFFR^+^/CD40^+^/TACI^+^), short-lived plasmablasts (CD19^+^/BCMA^+^/CD38^+^/BAFFR^+^/CD40^+^/TACI^+^), or long-lived plasma cells (BCMA^+^/CD38^+^/CD138^+^/BAFFR^+^/CD40^+^/TACI^+^) (176). Long-lived plasma cells are crucial components of immune memory.

Upon differentiation, B cells express unique surface molecules that serve as potential therapeutic targets for B cell-mediated diseases such as SLE. CD19, a key target for B cell depletion in CAR-T therapies, is expressed throughout multiple B cell stages-from pro-B cells to plasmablasts-except for plasma cells. The multifunctional role of CD19 in B cell activation, maturation, and signaling renders it an attractive target for B cell-directed therapies in SLE patients. CD20 and CD22 are expressed in B cells spanning the pre-B cell stage to memory B cells, yet are absent in plasmablasts and plasma cells. Other B cell receptors, including BCMA, CD38, and CD138, are extensively expressed in plasma cells. Additionally, BCMA and CD38 are also expressed in plasmablasts and plasma cells, thus targeting a broader spectrum of the B cell lineage. Consequently, therapeutic agents targeting these receptors can selectively target a specific subset and multi-subsets of the B cell lineage.

## 6 Preclinical studies of CAR-T cell therapy in SLE

Recent preclinical studies underscore the potential of CAR-T therapy for achieving remission in SLE and other autoimmune diseases, necessitating further investigation to optimize safety and efficacy. CD19 CAR-T cells featuring either CD28 or 4-1BB costimulatory motifs were evaluated in MRL-lpr mice, a model that develops an autoimmune disease resembling SLE. CD19 CAR-T cells not only effectively prevented disease pathogenesis prior to symptom onset but also demonstrated a more sustained B-cell depletion effect and a slight additional reduction in plasma cells, potentially distinguishing them from conventional B-cell depleting therapies and highlighting their clinical utility (173). Dr. Kansal R and colleagues demonstrated that treatment with CAR-T cells resulted in decreased concentrations of anti-DNA autoantibodies to undetectable levels in two different animal models. This corresponded with improved survival in mice, resolution of proteinuria, reduction of splenomegaly, and improved skin disease (172). Additionally, CAR-T cell-treated mice exhibited a differential T cell phenotype in peripheral blood and a distinct serum proteome, characterized by reduced levels of S100-A10, cathepsin D, tissue factor pathway inhibitor, and complement C4-B, while C3 levels were elevated. It is important to note that the beneficial effects of CAR-T cells in lupus nephritis observed in animal models are not consistent. For instance, in the research by Jin X and colleagues, no significant differences were observed in the levels of anti-dsDNA or in the histologic glomerular scores (172). Furthermore, CD19 CAR-Tregs were evaluated in a humanized mouse model of SLE *in vivo*. The CAR-Treg treatment resulted in a delayed onset of B-cell lymphopenia and restoration of the normal immune system composition in the inflamed organs, without observed toxicity or reprogramming towards Th17 pro-inflammatory cells (https://ashpublications.org/blood/article/142/Supplement%201/6813/506233).

## 7 Clinical studies of CAR-T cell therapy in SLE

SLE involves dysregulated B cells and plasma cells, and CAR-T cells provide a potent mechanism for depleting these cells and inducing drug-free and sustained remission, particularly in refractory cases where conventional treatments fail (177). Refractory SLE denotes cases in which the disease persists despite aggressive conventional therapies, including immunosuppressive agents and biologic treatments, with affected patients frequently experience severe disease progression involving vital organs, such as the kidneys, lungs and central nervous system. Therefore, the inclusion criteria for CAR-T therapy involves severe and active SLE that has failed to respond to multiple conventional therapies (with a median of 4-7 failed therapies in some trials), active organ involvement, and high disease activity scores, such as the SLE Disease Activity Index (SLEDAI) or the British Isles Lupus Assessment Group (BILAG). Prior to apheresis (APH) and CAR-T administration, it is essential for patients to discontinue immunosuppressive medications in order to maintain the quality of autologous T cells for effective CAR-T production and expansion in patients (177–179). However, due to safety concerns, patients with life-threatening infections or significant comorbidities that would contraindicate the use of CAR-T therapy, or those who are not suitable for lymphodepletion, may be excluded (179). Additionally, CAR-T cell therapies, particularly those targeting CD19, have shown promise in treating refractory SLE. Conditioning chemotherapy is essential for lymphodepletion, enabling optimal expansion and function of CAR-T cells. For example, cyclophosphamide and fludarabine are commonly employed and have been shown to create a “cytokine sink” to support CAR-T expansion and persistence. However, these regimens may independently modulate SLE activity due to their immunosuppressive effects. Nevertheless, controlled studies that are able to differentiate between chemotherapy effects and CAR-T effects are currently lacking.

In this section, we provide a comprehensive review of the latest clinical outcomes in both mono- or bi-specific CAR T-cell development for SLE, with a focus on clinical trials targeting B cells or plasma cells. **Table 1** represents an overview of CAR-T clinical trials in SLE.

**Table 1:** Summary of clinical trials using CAR T cell types for the treatment of SLE.

Clinical Trials.gov ID	Drug	Target	Status	Clinical Phase	Sponsor
NCT03030976	Anti-CD19-CAR-T cells	CD19	Unknown	Phase 1	Shanghai GeneChem Co., Ltd.
NCT05798117	YTB323	CD19	Recruiting	Phase 1/2	Novartis Pharmaceuticals
NCT05765006	Relma-cel	CD19	Recruiting	Phase 1	Shanghai Ming Ju Biotechnology Co., Ltd.
NCT06106906	UHCT230444	CD19	Recruiting	Phase 1/2	Wuhan Union Hospital, China
NCT05930314	CNCT19 cell injection	CD19	Recruiting	Early Phase I	Peking Union Medical College Hospital
NCT06150651	CAR T-cell therapy	CD19	Not yet recruiting	Phase 1	Chulalongkorn University
NCT06121297	CABA-201	CD19	Recruiting	Phase 1/2	Cabaletta Bio
NCT05869955	CC-97540	CD19	Recruiting	Phase 1	Juno Therapeutics, Inc.
NCT05988216	BRL-301	CD19	Recruiting	Not Applicable	Bioray Laboratories
NCT06189157	MB-CART19.1	CD19	Not yet recruiting	Phase 1/2	Miltenyi Biomedicine GmbH
NCT06222853	Anti-CD19-CAR-T cells	CD19	Recruiting	Phase I	The Children's Hospital of Zhejiang University School of Medicine
NCT06333483	Obe-cel	CD19	Recruiting	Phase I	Autolus Limited
NCT06340490	RJMty19	CD19	Not yet recruiting	Phase I	Guangdong Ruishun Biotech Co., Ltd
NCT06373991	ATHENA CAR-T	CD19	Recruiting	Phase I	EdiGene Inc.
NCT06153095	IMPT-514	CD19/CD20	Recruiting	Phase 1/2	ImmPACT Bio
NCT06249438	C-CAR168	CD20/BCMA	Recruiting	Phase I	RenJi Hospital
NCT06350110	CD19- BCMA CAR-T	CD19/BCMA	Not yet recruiting	Phase 1/2	Essen Biotech
NCT05474885	BCMA-CD19 cCAR T	CD19/BCMA	Recruiting	Phase 1	iCell Gene Therapeutics
NCT05030779	CD19/BCMA-001	CD19/BCMA	Unknown	Early Phase 1	Zhejiang University
NCT05858684	GC012F	CD19/BCMA	Recruiting	Early Phase 1	RenJi Hospital
NCT05846347	GC012F	CD19/BCMA	Recruiting	Phase 1	Zhejiang University

Data sourced from the https://clinicaltrials.gov/.

- CD19-Specific CAR-T: Dr. Georg Schett and colleagues initially described the successful application of CD19-targeted CAR-T cells in a 20-year-old woman suffering from severe, refractory SLE (178). Subsequently, they published further clinical data involving five additional patients with refractory SLE who underwent treatment with autologous anti-CD19 CAR-T cells (180). T cells were genetically modified with an anti-CD19 CAR vector, expanded, and subsequently reinfused into the patients at a dosage of 1.0 x 10^6 CAR-T cells per kilogram of body weight, following lymphodepletion induced by fludarabine and cyclophosphamide. In all five patients, CAR-T cells rapidly proliferated, ranging from 11% to 59% of total circulating T cells. This resulted in the complete elimination of B cells post-CAR-T cell therapy. Four of the five patients achieved a SLEDAI-2K score of zero, while patient 2 had a score of 2 at 3 months. Meanwhile, all patients showed normalization of complement factor levels and a decrease in anti-dsDNA antibody levels below the cutoff, achieving drug-free remission. In addition, all patients experienced either no or only mild cytokine release syndrome (CRS). None of the patients developed immune effector cell-associated neurotoxicity syndrome (ICANS) suggesting that this toxicity of CAR T-cell treatment may be less pronounced in patients with SLE. Overall, CD19 CAR-T cells effectively eliminate autoreactive B cells, leading to drug-free remission in patients with SLE. Notably, this remission was maintained even after B cell reconstitution. Recently reported monocentric clinical data from 15 patients, including 8 with SLE, detail their treatment with CD19-targeted CAR-T cells for refractory systemic autoimmune diseases (181). Patients underwent apheresis on day -13, followed by the initiation of lymphodepletion with intravenous fludarabine at 25 mg/m^2/day from days -5 to -3, and then with cyclophosphamide at 1000 mg/m^2/day on day -3. On day 1, patients received a single dose of 1.0 x 10^6 CD19-targeted CAR T cells per kilogram of body weight. As anticipated, the CAR-T cells rapidly expanded, and CD19^+^ B cells were quickly eliminated from the peripheral blood following the infusion. Additionally, 8 SLE patients achieved complete remission after three months and have maintained a SLEDAI-2K score of 0 since then. Five SLE patients, followed for 14-24 months, maintained remission despite B cell reconstitution. Moreover, complement factor C3 stayed within the normal range, proteinuria remained absent or low, and autoantibody seroconversion continued. The B cell count gradually increased, predominantly with a naïve phenotype. In summary, CD19 CAR-T cell therapy is a feasible, safe, and effective treatment for SLE.
  On May 30, 2024, at the EULAR 2024, JW (Cayman) Therapeutics Co., Ltd. presented clinical data for Relma-cel injection, a CD19-targeted CAR-T cell therapy, in patients with active SLE. As of December 18, 2023, three patients in the low-dose group (2.5x10^6 cells) received a single CAR-T cell infusion and completed at least four months of follow-up, demonstrating sustained clinical improvements. SLEDAI scores for these patients decreased from baseline scores ranging from 8 to 14 to 0 or 1. All patients achieved a SLE Response Index-4 (SRI-4), and two also met the Lupus Low Disease Activity State (LLDAS) criteria. Moreover, the safety profile of Relma-cel was encouraging; two patients experienced CRS, and no ICANS were observed. The ongoing investigator-initiated trial (IIT) indicates that 100% of patients who have received Relma-cel and completed at least three months of follow-up have achieved an SRI-4 response. Notably, four patients followed for up to six months have maintained their response. Following infusion, 91.67% of the 11 patients discontinued traditional corticosteroids and immunosuppressants, easing the medication burden and potential side effects. Additionally, significant improvements in organ damage, SLE disease activity, anti-dsDNA antibody levels, and urinary protein excretion were observed. All patients achieved rapid peripheral B-cell depletion after infusion, with a median time to depletion of four days. These preliminary data suggest that Relma-cel could offer profound and enduring disease remission in patients with moderate-to-severe SLE, featuring a favorable safety profile that warrants further investigation.
- CD19/BCMA-Specific CAR-T: Dr. He Huang and colleagues reported the findings of a Phase I clinical trial in which 12 patients with refractory SLE were treated with CD19/BCMA CAR-T cells, according to data presented at the 2023 ASH meeting (https://ash.confex.com/ash/2023/webprogram/Paper186669.html). Participants underwent lymphocyte-depleting chemotherapy with cyclophosphamide and fludarabine, administered from day -4 to day -2, preceding the sequential infusion of CD19 and BCMA CAR-T cells (at a dosage of 1.0-2.0 x 10^6 cells/kg) on day 0. Three patients received the lower dosage of 1.0 x 10^6 cells/kg for each cell type, while the remaining nine received the higher dosage of 2.0 x 10^6 cells/kg. As of July 31, 2023, BCMA CAR-T cells exhibited expansion in all patients; however, CD19 CAR-T cell expansion was not detected in one patient. Following CAR-T cell expansion, there was a notable depletion of circulating B cells. The SLEDAI-2K scores indicated a marked reduction, with the average score decreasing from 18.3 to 1.5 across all patients. Although low-level proteinuria persisted in some cases, this was likely due to previous glomerular damage. Regarding safety, all patients experienced grade 1 CRS, with no instances of ICANS. Hematotoxicity was grade 4 in 11 patients (91.7%) and grade 3 in one patient (8.3%) and was clinically manageable in all cases. Finally, B cell recovery was observed approximately three months post CAR-T therapy.

Dr. Weijia Wang and colleagues conducted a clinical study involving 12 patients with SLE and LN who underwent bi-specific BCMA-CD19 compound CAR-T (cCAR-T) therapy, targeting B cells and plasma cells (33). All patients discontinued medication use after apheresis and prior to cyclophosphamide/fludarabine conditioning. Patients received a single infusion of 3.0 x 10^6 cCAR-T cells per kilogram of body weight on day 0. In terms of efficacy, plasma cells were completely eradicated within the first month. The levels of complement factors C3 and C4 normalized within 21 days. 11 of 13 patients experienced significant symptom relief within the first month, without requiring additional medication. Moreover, three patients maintained symptom-free status and medication-free remission for over a year (up to 44 months). In addition, LN patients experienced significant renal function improvement within the first 6 months.

In terms of safety, cCAR-T therapy showed good tolerability, with no cases of CRS or ICANs, and no significant infectious complications were observed, meeting the specific requirement for infectious safety outcomes associated with the bi-specific BCMA/CD19 compounds. Patients were closely monitored for IgA and IgG levels, and received IVIG when necessary. The white blood cell (WBC) count normalized within 7-21 days. B cell counts reached baseline levels in all patients, with an average duration of 90 days (range: 40 to 150 days). All patients treated for more than 150 days exhibited normal levels of IgM. Given the normalization of B cells and IgM levels within 150 days, full restoration of humoral immunity is anticipated for all patients. Overall, cCAR-T cells effectively eliminate all autoantibodies, reconstitute the B cells, reset humoral immune systems, and induce long-term remission without further medication.

In addition to evaluating the efficacy and safety of CAR-T therapy for SLE, recent studies have also investigated immunological changes in patients to better understand the impact of CAR-T cells. Nunez et al. analyzed the serum of six refractory SLE patients treated with CAR-T cells for inflammatory cytokines. The results revealed an increase in B cell homeostatic cytokines IL-7 and BAFF, along with a decrease in IL-6, IL-10, and tumor necrosis factor (TNF)-α, three months post-CAR-T cell infusion in all six patients. Anti-DNA antibodies, used for diagnosing SLE and correlating with disease severity, were not reduced in one out of six patients under CAR-T cell treatment, suggesting that larger studies with longer follow-up periods are necessary to fully understand the effects of CAR-T therapy on SLE (182). Furthermore, Wang W et al. demonstrated that post-BCMA-CD19 compound CAR-T cells (cCAR) led to symptom and medication-free remission (MFR) and complete B cell recovery within 2-6 months. However, one patient experienced a relapse and required a second therapeutic infusion, necessitating the resumption of immunosuppressive therapy, likely due to the presence of anti-CAR antibodies (33). The essential role of autoreactive B cells in SLE pathogenesis is accompanied by a B cell-dependent increase in type-I IFN signaling, as noted by Wilhelm A et al. This underscores the potential for developing novel co-therapies, such as blocking IFN signaling and depleting B cells, to efficiently and rapidly suppress inflammation and reset autoimmunity (183).

## 8 Challenges

Technological advancements and compelling evidence indicate that CAR-T cell therapy is promising for treating SLE (30, 31). However, the implementation of this innovative strategy in clinical practice requires thorough preclinical and clinical research to overcome the numerous challenges. The foremost challenge in CAR-T therapy for SLE is its limited targeting scope. Considering SLE’s complexity, with its multiple autoantigens and immune pathways, current CAR-T technologies, often targeting a single antigen or pathway, may not fully address the disease’s complexity, possibly neglecting some pathogenic factors (15, 16). By targeting CD19^+^ B lymphocytes, CD19-specific CAR-T becomes an emerging treatment for SLE. With the limited clinical trials and follow up, CD19-specific CAR-T therapy outcomes for blood cancers and SLE across key metrics including persistence, expansion, B cell eradication and relapse were compared. In B-cell malignancies, CD19-specific CAR-T cells exhibit robust expansion and strong persistence which correlates with long-term remission but also increases the risk of toxicities like CRS, neurotoxicity and durable B cell aplasia (184). In SLE, CD19-specific CAR-T cell can rapidly deplete B cells and persist shorten. The reduced CAR-T persistence in SLE aligns with faster B cell recovery, may attribute to fewer adverse effects, reduced inflammation and remission without inducing severe immune suppression. Moreover, improper dosing regimens could heighten risks for SLE patients. Although CAR-T therapies aim to boost immune responses against specific targets, there is a concern that their use in a dysregulated immune system might exacerbate existing pathological conditions or result in unforeseen side effects. Furthermore, the efficacy and duration of CAR-T therapy differ among patients, with some exhibiting a limited response. Finally, the high cost and limited availability of CAR-T therapy, a personalized approach reliant on sophisticated technology and intricate manufacturing processes (185), may impede the widespread application of this treatment among SLE patients.

## 9 Perspectives

**Figure 5:**
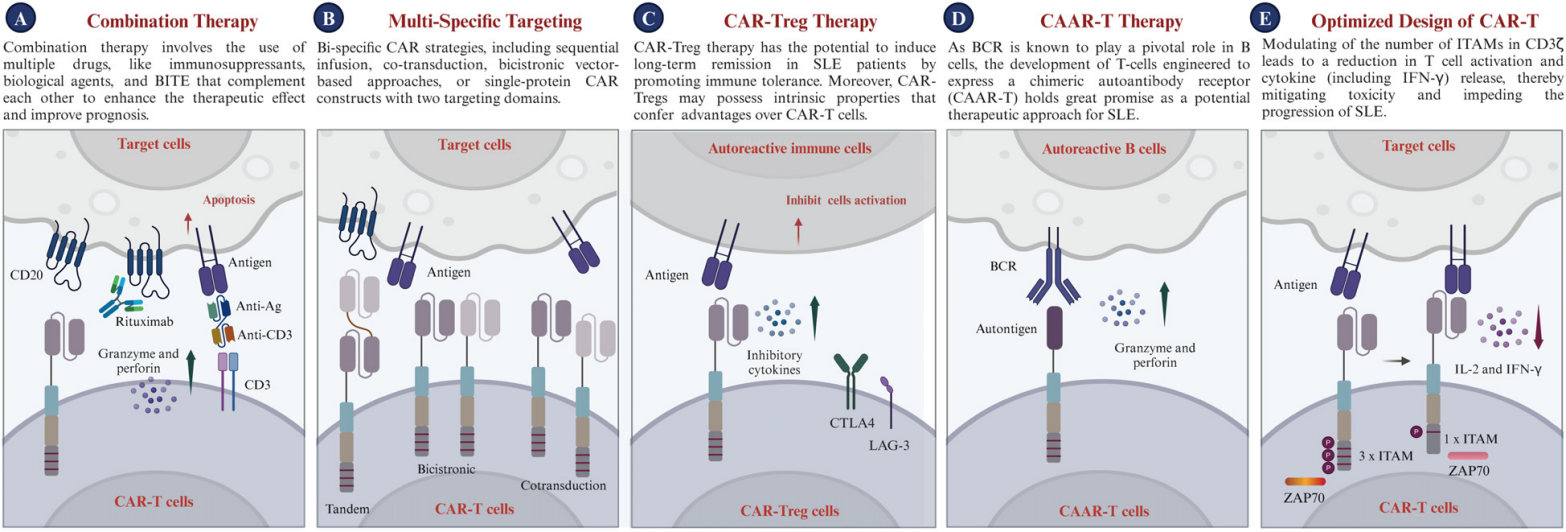
Prospects of CAR-T therapy in clinical applications. **(A)** Combination Therapy: Utilizes synergistic combinations of immunosuppressants, biological agents, or BiTEs to enhance efficacy and improve patient prognosis, while minimizing toxicity. **(B)** Bi-specific Targeting: Enables the expression of two distinct chimeric antigen receptors (CARs) within a single T cell or across different T cells, engineering the CAR-T cells to recognize multiple antigens and target various cell populations. **(C)** CAR-Treg Cell Therapy: Introduces CAR-Treg cells targeting autoreactive immune cells, utilizing inhibitory cytokines and immune checkpoint pathways (CTLA4, LAG-3) to suppress activation and induce immune tolerance. **(D)** CAAR-T Cell Therapy: Highlights the potential of chimeric autoantibody receptor T cells (CAAR-T cells) to target autoreactive B cells via the B cell receptor (BCR), offering a promising therapeutic strategy for SLE. **(E)** Optimized CAR design for SLE: Schematic diagram of the molecular composition of CAR-T cells, including signaling domains such as CD3ζ with varying immunoreceptor tyrosine-based activation motifs (ITAMs), which modulate T cell activation and cytokine release, crucial for managing treatment-related toxicity. CTLA4, Cytotoxic T lymphocyte antigen 4; LAG-3, Lymphocyte activation gene 3; IL-2, Interleukin-2; IFN-γ, Interferon-γ; ITAM, Immunoreceptor tyrosine-based activation motif.

Despite its demonstrated potential in treating SLE, future research on CAR-T therapy should concentrate on refining targeting mechanisms, mitigating safety risks, enhancing therapeutic efficacy, lowering preparation or medical costs, and creating universally effective treatments to maximize the clinical utility of CAR-T in SLE. In this section, we discuss the potential future applications of CAR-T therapy for the treatment of SLE (**Figure 5**).

- Combination Therapy: Combination therapy employs a variety of drugs, including immunosuppressants and biological agents, which work synergistically to enhance treatment efficacy and improve patient outcomes without amplifying toxicity (186, 187). The concept is particularly appealing for SLE treatment because it promises to modulate the overactive immune response, reduce the likelihood of complications from therapy, and improve overall safety. Additionally, the combination of CAR-T therapy with biological agents such as Belimumab could potentially accelerate autoantibody clearance and suppress B cell activation prompted by BAFF (128, 188). Moreover, recent studies have underscored the potential of bispecific T cell engagers (BiTEs) in treating autoimmune diseases (189, 190). BiTEs are a targeted immunotherapeutic platform that directs patients’ own T cells to target cells, thereby enabling the elimination of B cells through T cell engagement (191). Blinatumomab, a BiTE targeting CD3 and CD19, has shown efficacy in treating patients with rheumatoid arthritis (RA); six patients with multidrug-resistant RA observed B cell depletion and a swift reduction in clinical disease activity (189). Furthermore, teclistamab, a BiTE targeting CD3 and BCMA, has been reported to improve disease activity in patients with autoimmune diseases, including cases of severe, refractory SLE (190). Synergy among these diverse treatments could provide more effective options for managing SLE and maintaining long-term control of autoimmune activity. In the long term, the implementation of such strategies may pave the way for the development of safe and effective therapies for SLE. Nonetheless, thorough research, stringent clinical trials, and a deep comprehension of disease pathogenesis are essential for steering the future integration of CAR-T with other therapeutic strategies in SLE.
- Multi-specific targeting CAR-T: Multi-specific CAR strategies encompass sequential infusion, co-transduction, bicistronic vector-based approaches, and single-protein CAR constructs featuring multiple targeting domains (192). The primary advantage of multi-specific CAR-T therapy for SLE lies in its ability to target multiple relevant antigens simultaneously, enhancing precision in addressing SLE’s complex and heterogeneous nature (193, 194). Meanwhile, multi-specific CAR-T therapy can eliminate autoreactive T cells and regulate a broader immune response. This multi-action approach aims to restore immune balance and alleviate the autoimmune response by specifically targeting pathogenic cells and delivering regulatory signals. Additionally, targeting long-lived plasma cells, which produce diverse autoantibodies, contributes to SLE management. Thus, targeting autoreactive immune cells through various receptors could offer a novel and promising approach for treating SLE (33), in addition to CD19 CAR-T therapy. The potential application of multi-specific CAR-T therapy for SLE holds significant promise for the future.
- CAR-Treg Therapy: CAR-Treg cells demonstrate significant potential for the treatment of SLE (195). A recent investigation demonstrated that engineered CAR-Tregs can effectively suppress immune responses associated with SLE, specifically by targeting autoreactive B cells. In a humanized mouse model of lupus, CAR-Tregs delayed B cell lymphopenia and modulated immune responses without inducing toxicity (196). They are engineered to express chimeric antigen receptors and signaling domains associated with Tregs, primarily harnessing the regulatory capabilities of T-regulatory cells (197). Tregs, a distinct subset of T cells, are known for their key role in modulating immune responses. Tregs inhibit inflammatory responses and regulate autoreactive immune cells, essential for maintaining immune balance and preventing autoimmunity (198). CAR-Treg therapy holds the potential to induce long-term remission in SLE patients by promoting immune tolerance through the action of Tregs. Moreover, the inherent properties of Tregs give CAR-Tregs unique advantages over traditional CAR-T cells. While CAR-T cells target specific cells for elimination, CAR-Tregs are designed to regulate the immune system, inhibiting autoreactive B and T cells implicated in SLE development. Overall, CAR-Treg cell therapy, with its potential to improve cell-based therapy efficacy and avoid broad immunosuppression, offers a novel, targeted approach for SLE, potentially transforming treatment strategies for this intricate condition.
- Chimeric autoantibody receptor T Therapy: As autoantibodies are known to play a pivotal role in the progression of SLE, the development of T-cells engineered to express a chimeric autoantibody receptor (CAAR-T) holds great promise as a potential therapeutic approach for these autoimmune diseases (17, 199). Specifically, T cells were genetically modified to express a CAAR, which consists of a hinge and transmembrane domain connected to an intracellular activation domain that is identical to the traditional second-generation CARs (200). CAAR-T cells selectively target B lymphocytes expressing specific autoantibodies based on their BCR specificity, thereby inhibiting autoantibody production and preventing autoantigen presentation to T cells. This selective targeting, which does not affect the total B cell population, could potentially reduce the autoimmune response and associated inflammation without causing broad immunosuppression, which is a common side effect of current treatments. Preclinical studies have shown that CAAR-T cells are safe and effective in treating autoimmune diseases (201). Hence, CAAR-T cells may offer a platform technology for B-cell-mediated autoimmune diseases, with their use in SLE treatment potentially introducing an innovative and targeted therapeutic approach. This could significantly transform the management of this complex autoimmune disorder. However, in diseases like pemphigus, the absence of intercellular antigens in the lower epidermal layers of pemphigus foliaceus leads to unique blister localization, posing challenges for antigen-specific therapies (202, 203). Additionally, the limitation of CAAR therapy is its specific targeting of autoantigens, whereas SLE involves multiple autoantibody responses against various targets, including DNA, RNA, ribonucleoproteins, and phospholipids. The diversity of autoantigens and patient heterogeneity pose challenges for a single CAAR therapy to effectively address the full spectrum of immune dysregulation (204).
- Optimized design of CAR-T: Given the widespread interest in CAR-T cell-based therapies, researchers have focused on developing innovative CAR structures to improve their functionality, inhibit the advancement of SLE, and mitigate adverse effects (36). The CAR design encompasses four major modules: binder, spacer, transmembrane domain, and cytoplasmic domain. Humanized binders mitigate immunogenicity, enhancing the safety and persistence of CAR-T therapies *in vivo (* 205). Appropriate spacer domains are crucial for optimal target recognition and immune synapse formation (206). Differences in transmembrane domains can regulate the functional strength of CAR-T cells without impairing the antigen-binding properties of the binders and the signaling properties of T cells (206). Thus, the selection of appropriate spacer and transmembrane domains depends on the cell type and target. Additionally, immunoreceptor Tyrosine Activation Motifs (ITAMs) in the TCR-CD3 complex’s cytoplasmic domains are key phosphorylation sites essential for initiating signaling (207). In certain cases, modulating the number of ITAMs in CD3ζ or employing other TCR subunits such as CD3δ, CD3γ, or CD3ϵ can regulate T cell activation and IFN-γ release, thereby mitigating toxicity and impeding the progression of SLE (208).
- Universal CAR-T/NK (IPS-T/UCAR-T/NK): The development of universal CAR-T/NK (UCAR-T/NK) cell therapy represents an attractive breakthrough, potentially overcoming many limitations associated with conventional CAR-T/NK (209). UCAR-T/NK, also known as “off-the-shelf” CAR-T/NK, offers unique advantages over traditional personalized CAR-T/NK therapies (210). Firstly, the off-the-shelf nature of UCAR-T/NK eliminates the need for individual customization for each patient, significantly reducing the preparation time from manufacturing to the clinic and potentially improving treatment accessibility. Secondly, the standardized manufacturing of UCAR-T/NK reduces costs, increasing its economic viability and suitability for broad clinical use. Additionally, universal CAR-T/NK exhibits superior repeatability thanks to its standardized manufacturing method, which ensures consistent treatment outcomes. Furthermore, the logistics and management of universal CAR-T/NK are simplified as they can be prepared in advance and stored, thereby streamlining treatment complexity. While UCAR-T/NK cells may exhibit reduced *in vivo* persistence compared to autologous CAR-T/NK cells, future repeated infusion of CAR-T/NK could address SLE recurrence (210, 211). Overall, the application of universal CAR-T/NK cells in SLE holds significant promise due to their versatility, large-scale manufacturing capabilities, cost-effectiveness, and reproducibility.

## 10 Conclusions

**Figure 6:**
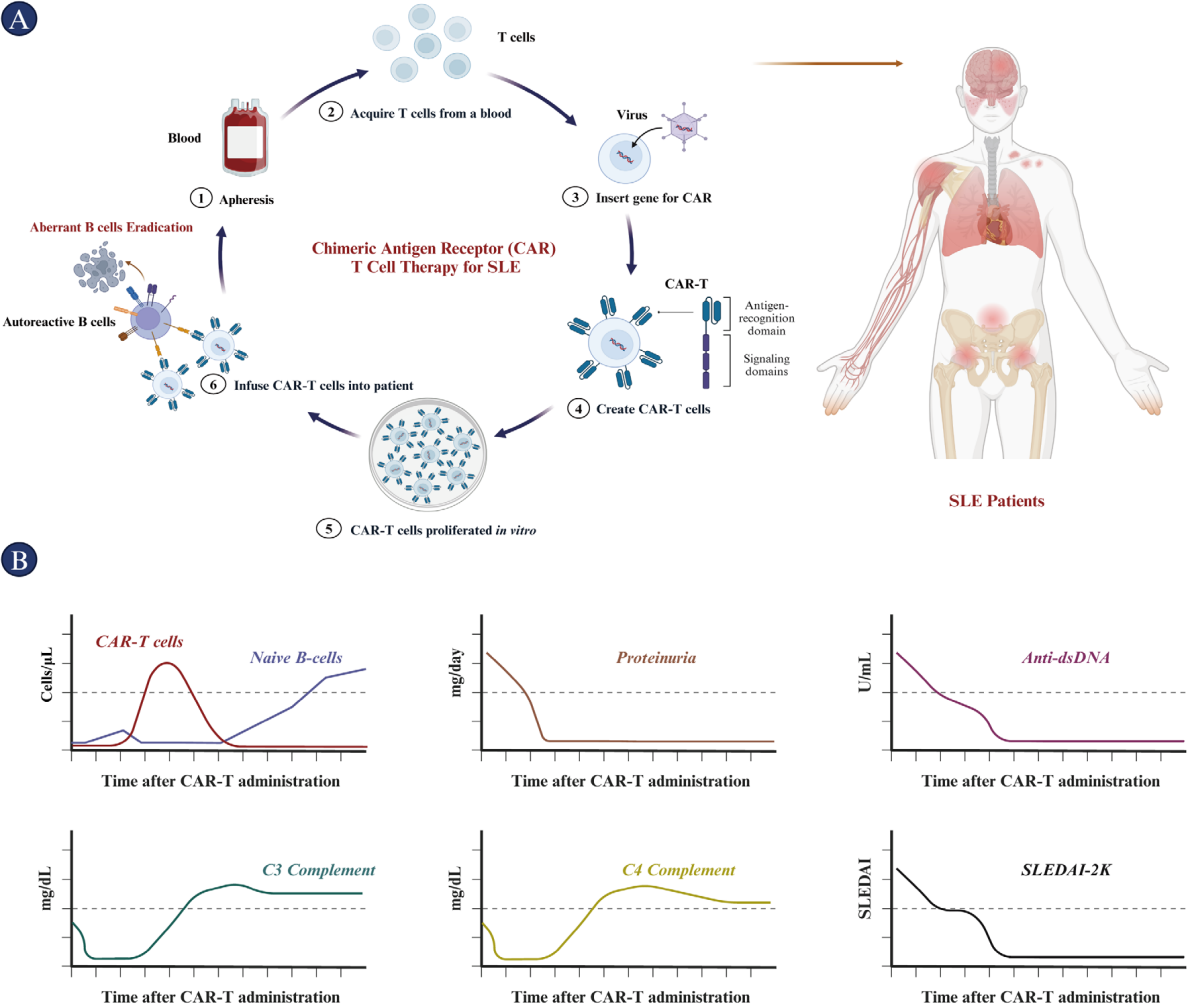
Procedure and outcomes of CAR-T cell treatment in SLE patients. **(A)** The CAR-T cell therapy procedure for SLE includes the isolation and purification of T cells from the patient. These T cells are then prepared for CAR-T cell therapy through lentiviral transfection, followed by *in vitro* proliferation. Subsequently, the CAR-T cells are infused intravenously into patients preconditioned with cyclophosphamide and fludarabine. **(B)** The immunological and clinical features of patients post-CAR-T cell therapy are depicted from top to bottom: CAR-T cell and naïve B cell counts, proteinuria, anti-dsDNA antibody levels, C3 complement protein levels, C4 complement protein levels, and SLEDAI scores over time. CAR, chimeric antigen receptor; dsDNA, double-stranded DNA; SLEDAI, Systemic lupus erythematosus disease activity index.

SLE, a complex autoimmune disease, is characterized by autoantibody production and immune system dysfunction, leading to organ damage (15–17). Advancements in therapeutic strategies have markedly improved patient outcomes since the mid-20th century, attributed to corticosteroids and immunosuppressive agents (105, 106, 110, 111). Recent studies increasingly show the pivotal role of abnormal B-cell or plasma-cell activation in SLE, making B-cell or plasma-cell targeted therapies a promising strategy (24). Despite the development of many monoclonal or bi-specific antibodies targeting B cells, their efficacy is still limited with a high relapse rate (25, 26). CAR-T cell therapy represents a new era in treating SLE (**Figure 6**), as its prolonged *in vivo* persistence offers a new horizon for achieving sustained remission and potentially curing the disease, as demonstrated by clinical trials (30, 31). Potential applications of CAR-T cell therapy in SLE include targeting B cells, plasma cells, and autoantigens, modulating immune homeostasis, and reduction of disease activity (31). Hence, this article provides a comprehensive exploration regarding the rationales and applications of CAR-T in treating SLE, clarifies the critical immunological features of the disease, summarizes the clinical safety and efficacy of this approach in eliminating B cells and plasma cells in SLE or LN patients, and highlights the limitations of current CAR-T therapy. Moreover, this article offers insights for guiding novel CAR-T development and optimization, presenting an overview of the advancements, unmet medical needs, and potential applications associated with using either autologous or universal CAR-T cells as a therapeutic strategy in addressing SLE and LN. The continued pursuit of preclinical and clinical investigations is crucial to refine and validate CAR-T cell therapy, which will bring us closer to a new era in the treatment of SLE or other relevant autoimmune diseases.
